# A Clinical Diagnostic Model for Predicting Influenza among Young Adult Military Personnel with Febrile Respiratory Illness in Singapore

**DOI:** 10.1371/journal.pone.0017468

**Published:** 2011-03-02

**Authors:** Vernon J. Lee, Jonathan Yap, Alex R. Cook, Chi Hsien Tan, Jin-Phang Loh, Wee-Hong Koh, Elizabeth A. S. Lim, Jasper C. W. Liaw, Janet S. W. Chew, Iqbal Hossain, Ka Wei Chan, Pei-Jun Ting, Sock-Hoon Ng, Qiuhan Gao, Paul M. Kelly, Mark I. Chen, Paul A. Tambyah, Boon Huan Tan

**Affiliations:** 1 Biodefence Centre, Ministry of Defence, Singapore, Singapore; 2 Centre for Health Services Research, National University of Singapore, Singapore, Singapore; 3 Department of Epidemiology and Public Health, National University of Singapore, Singapore, Singapore; 4 National Centre for Epidemiology and Population Health, Australian National University, Canberra, Australia; 5 Department of Statistics and Applied Probability, National University of Singapore, Singapore, Singapore; 6 Department of Clinical Epidemiology, Tan Tock Seng Hospital, Singapore, Singapore; 7 Defence Medical and Environmental Research Institute, DSO National Laboratories, Singapore, Singapore; 8 Division of Infectious Diseases, National University of Singapore, Singapore, Singapore; University of Hong Kong, Hong Kong

## Abstract

**Introduction:**

Influenza infections present with wide-ranging clinical features. We aim to compare the differences in presentation between influenza and non-influenza cases among those with febrile respiratory illness (FRI) to determine predictors of influenza infection.

**Methods:**

Personnel with FRI (defined as fever≥37.5°C, with cough or sore throat) were recruited from the sentinel surveillance system in the Singapore military. Nasal washes were collected, and tested using the Resplex II and additional PCR assays for etiological determination. Interviewer-administered questionnaires collected information on patient demographics and clinical features. Univariate comparison of the various parameters was conducted, with statistically significant parameters entered into a multivariate logistic regression model. The final multivariate model for influenza versus non-influenza cases was used to build a predictive probability clinical diagnostic model.

**Results:**

821 out of 2858 subjects recruited from 11 May 2009 to 25 Jun 2010 had influenza, of which 434 (52.9%) had 2009 influenza A (H1N1), 58 (7.1%) seasonal influenza A (H3N2) and 269 (32.8%) influenza B. Influenza-positive cases were significantly more likely to present with running nose, chills and rigors, ocular symptoms and higher temperature, and less likely with sore throat, photophobia, injected pharynx, and nausea/vomiting. Our clinical diagnostic model had a sensitivity of 65% (95% CI: 58%, 72%), specificity of 69% (95% CI: 62%, 75%), and overall accuracy of 68% (95% CI: 64%, 71%), performing significantly better than conventional influenza-like illness (ILI) criteria.

**Conclusions:**

Use of a clinical diagnostic model may help predict influenza better than the conventional ILI definition among young adults with FRI.

## Introduction

Influenza infections result in a wide range of clinical presentations, from the classical influenza-like illness (ILI), to milder respiratory infections, and subclinical infections. Determining the clinical predictors of influenza infection is important for the diagnosis and management of patients presenting with respiratory illness, helping to guide appropriate antiviral therapy, and to avoid unnecessary antibiotic use. This is particularly important in the young adult population, which constitutes an economically productive age group whereby early treatment may reduce work absenteeism [1]. The recent 2009 H1N1 pandemic has shown that young adults have a higher infection rate compared to other age groups [2]. For essential public services such as the military, police, civil defence, and healthcare with substantial proportions of young adults, early recognition and treatment may reduce service disruptions.

There has been research describing the differences in symptoms between influenza and non-influenza cases. However, few have been performed in tropical countries, where a large proportion of the world's population reside. Influenza morbidity and mortality in tropical countries like Singapore has been shown to be comparable to temperate countries [3], [4]. Furthermore, there has also been substantial co-circulation of other etiologic agents that can similarly cause acute respiratory illnesses [5]. While two recent tropical studies sought to differentiate the symptoms of these clinical entities, they had only limited number of cases [6], [7], and were based only on hospital attendances in the peri-pandemic period, where inclusion criteria might be atypical.

Using data from a respiratory disease sentinel surveillance system in the Singapore military, we compare the differences in clinical presentation between influenza and non-influenza cases in young adults with febrile respiratory illness to determine predictors of influenza infection and aid case management especially where laboratory confirmation is not possible.

## Methods

Singapore is a city state in tropical South-East Asia with 5 million people, with all Singaporean males serving two years of military service after high school. These servicemen live in barracks-style accommodation during weekdays and return home during weekends, maintaining continued interaction between the military and the Singapore population.

The Singapore military began a sentinel respiratory disease surveillance program in 4 major camps, including a recruit training camp, on 11 May 2009 (epidemiological-week 19), just before community spread of pandemic H1N1 in late-June 2009 [8], [9]. All personnel who visited the primary healthcare clinics in these camps during the main consultation hours with febrile respiratory illness (FRI)—defined as the presence of fever ≥37.5°C with cough or sore throat—were recruited. The use of FRI contrasts with the usual measure of influenza-like illness (ILI, defined as fever ≥38.0°C with cough or sore throat); our choice reflected the desire to capture other febrile cases that also result in substantial absenteeism; while limiting cases to those with fever as an indicator of potential severity and absenteeism.

Repeat visits for the same illness episode as assessed by the consulting physician were excluded to avoid double counting. Nasal washes, collected separately from each side of the nose, were taken from consenting participants by trained medical staff, collected in viral transport media, and sent to the laboratory within 24 hours. Nasal washes were used as they have been shown to be equally or more sensitive than other methods such as nasal or throat swabs, and nasopharyngeal aspirates, in the detection of respiratory infections such as influenza [10]–[12].

In addition, interviewer-administered questionnaires were completed during the medical consultation, collecting information on patient demographics and clinical features. A follow-up phone questionnaire was conducted 2 weeks after the initial consultation to determine symptoms present during the entire course of illness.

Written informed consent was obtained. The study was approved by the military's Joint Medical Committee for Research, and by the institutional review boards of the National University of Singapore, and the Australian National University.

### Laboratory Methods

To determine the etiology, we used the multiplex PCR strategy based on the Resplex assays described below, and performed additional singleplex PCR assays to determine the influenza subtype.

Total nucleic acids were extracted from each specimen using the DNA minikit (Qiagen, Inc, Valencia, CA, USA) according to the manufacturer's instructions. Five µl of extract were tested with Resplex I and II (version 2.0, Qiagen, Inc., Valencia, CA, USA) for the presence of respiratory micro-organisms on the LiquiChip 200 Workstation, again according to the manufacturer's instructions. The Resplex I and II (version 2.0) assays are multiplex PCR assays coupled with bead array detection technology and can simultaneously detect and subtype 18 different viruses and bacteria including influenza A and influenza B [13]–[15].

Specimens that were Resplex II positive for influenza A were further subtyped with real-time PCR for H1 or H3 (Singapore Ministry of Health), or for pandemic H1N1 [16]. Briefly, five µl of total genetic extracts were tested with the one-step SuperscriptIII/Platinum Taq kit (Invitrogen, Carlsbad, CA, USA) following the manufacturer's instructions on either the LightCycler machine from Roche or the Applied Biosystems real-time PCR machine (7500).

### Statistical Analysis

We compared differences in overall clinical presentation between influenza and all non-influenza FRI cases. Univariate comparison of demographic parameters, symptoms and signs was conducted using logistic regression to determine statistically significant parameters of interest. Potential confounding was addressed by performing multivariate analyses where characteristics found to be statistically significant in univariate analyses were entered into a multivariate logistic regression model to identify independent clinical predictors, with non-significant terms in the multivariate analysis dropped one at a time starting with the highest *p*-value. To address another source of potential confounding among the remaining variables, we assessed for interactions between these variables but none proved significant. All statistical analyses were performed using Stata 9.0 (Stata Corp., College Station, TX, USA) and R (R Core Development Team). All tests were conducted at the 5% level of significance, with no explicit adjustment for multiple comparisons; instead, where appropriate, we present the expected number of false positive findings under the assumption that all null hypotheses are correct, a strongly conservative assumption. We report odds ratios (OR) and corresponding 95% confidence intervals (CI) where applicable.

The final multivariate model for influenza versus non-influenza cases was used to build a predictive probability equation as a clinical diagnostic model to determine the likelihood of influenza infection given the clinical characteristics. For this we developed the receiver operating characteristic (ROC) curve whence the area under the ROC (AUC) was calculated and two cut-off points determined: one maximizing the sum of sensitivity and specificity, the other maximising specificity while keeping sensitivity at 90%. Ten-fold cross-validation was used to guard against over-fitting, with AUC, sensitivity and specificity scores averaged over the ten folds.

## Results

A total of 2858 eligible subjects were recruited from 11 May 2009 to 25 Jun 2010. Of these 2858 subjects, 2717 (95.1%) completed the telephone follow-up. The average age was 21 years old (SD 3.2), and 2853 (99.8%) were male. Of the 2858 subjects, there were 821 influenza cases, of which 434 (52.9% of all influenza cases) were 2009 pandemic influenza A (H1N1), 58 (7.1%) seasonal influenza A (H3N2), 269 (32.8%) influenza B, and 10 (1.2%) seasonal influenza A (H1N1), with 6 co-infections and 44 unsubtypable.

There were a total of 70 influenza vaccine failures, defined as seasonal or pandemic influenza infections that occurred despite previous vaccination with the relevant seasonal or pandemic vaccine respectively. Of these, there were 43 pandemic H1N1 vaccine failures, although 27 (63%) were vaccinated less than 2 weeks before onset of symptoms; 11 H3N2 vaccine failures, and 16 influenza B vaccine failures. There were no statistically discernible differences in influenza severity (fever ≥38.0°C or breathlessness) for vaccine failures compared to other influenza cases.

**Figure 1:**
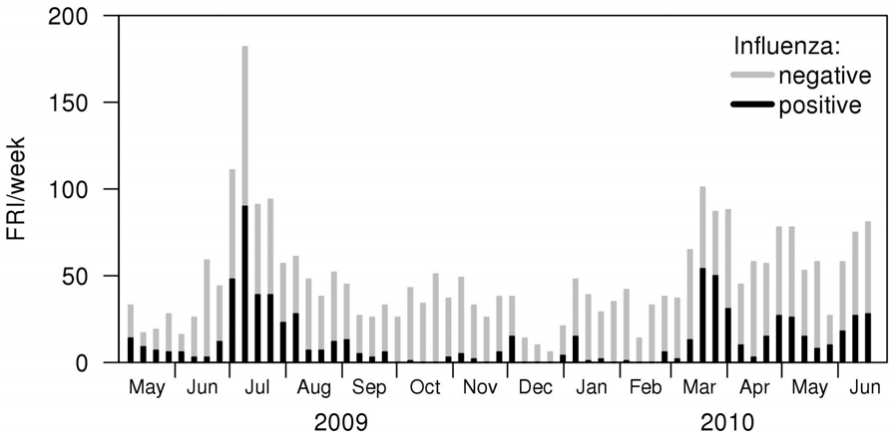
Weekly FRI cases, by influenza RT-PCR positivity, in 2009/10 in the Singapore military.

Figure 1 shows the number of FRI cases sampled per week, and the proportion of these cases that tested positive for influenza. For the non-influenza FRI cases, 289 (10.1% of all subjects) were diagnosed with coxsackie viruses/echoviruses, 247 (8.6%) rhinovirus, 217 (7.6%) H. influenzae, 130 (4.5%) coronaviruses, 76 (2.7%) parainfluenza viruses, 47 (1.6%) human metapneumovirus, 27 N. meningitidis, 12 S. pneumoniae, 5 adenoviruses, 2 RSV, and 1 bocavirus.

### Clinical Features

**Figure 2:**
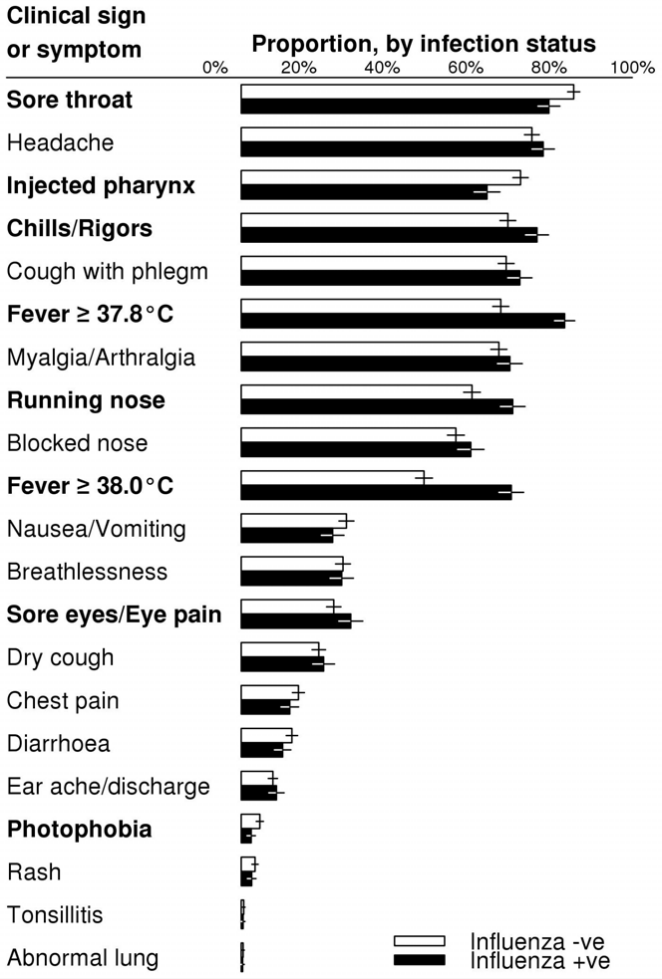
Univariate comparison of clinical signs or symptoms between influenza-positive and influenza-negative cases. Symptoms or signs are ranked by frequency for non-influenza cases. Empirical frequencies of presentation of each symptom or sign are presented in the right column as bars, with 95% confidence intervals represented by whiskers. Symptoms or signs that are statistically discernibly different at the 5% level are displayed in bold font. With 21 tests, the conservative expected number of false discoveries is 1.1.

Univariate analyses comparing the clinical features between influenza and non-influenza cases are presented in Figure 2, while the multivariate analyses adjusting for possible confounders are presented in Table 1.

**Table 1:** Multivariate analysis comparing clinical features of influenza-positive with all influenza-negative FRI cases.

Influenza Positive vs Negative*		
Parameters	Adjusted Odds Ratio (95% CI)	p value
Sore throat	0.62 (0.48, 0.82)	<0.001
Running nose	1.86 (1.52, 2.29)	<0.001
Chills/rigors	1.52 (1.20, 1.91)	<0.001
Photophobia	0.49 (0.29, 0.83)	0.007
Fever (≥37.8°C)Fever (≥38°C)	1.64 (1.19, 2.26)2.15 (1.65, 2.80)	0.003<0.001
Injected pharynx	0.69 (0.56, 0.86)	<0.001
Nausea/VomitingEye symptoms	0.74 (0.59, 0.92)1.25 (1.01, 1.55)	0.0070.04

*Age, sore throat, running nose, sore eyes or eye pain, chills/rigors, photophobia, Fever ≥37.8°C, Fever ≥38.0°C, and injected pharynx were included in the analysis before non-significant terms were sequentially removed. With nine tests, the conservative expected number of false discoveries is 0.45

From the univariate and multivariate analyses, influenza-positive cases were significantly more likely to present with running nose, chills and rigors, and higher temperature, and less likely to present with sore throat, photophobia, and injected pharynx, compared to influenza-negative cases (Figure 2 and Table 1). Ocular symptoms were significant on univariate but only marginally so on multivariate analysis, while nausea/vomiting was borderline significant on univariate but clearly significant on multivariate analysis. Based on the final model's maximum likelihood estimates, we created a diagnostic index that predicted influenza infection based on clinical presentation. The predicted probability of influenza infection *(p_i_)* was calculated as follows:

10ln 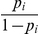 =  –31 – 5[sore throat] + 6[running nose] + 2[ocular symptoms] – 3[nausea/vomiting] + 4[chills/rigors] – 7[photophobia] + 5[fever≥37.8] + 8[fever≥38] – 4[injected pharynx]where [A]  = 1 if the patient presents with that symptom or sign and 0 otherwise. A score (on the right hand side) of 0 corresponds to a 50% chance of influenza infection, -10 to about a 25% chance, -5 to about a 40% chance. The fever terms are cumulative, i.e. a fever of 37.9 adds 5 to the score, while a fever of 38.2 adds 13.

The AUC under ten-fold cross-validation was 69% (95% CI: 61%, 76%). Using a cut-off to maximize sensitivity and specificity, the model had sensitivity of 65% (95% CI: 58%, 72%), specificity of 69% (95% CI: 62%, 75%), and overall accuracy of 68% (95% CI: 64%, 71%) under ten-fold cross validation. The model allows for differing cut-off specifications using the indicated criteria (Table 2). The relatively poor performance of ILI alone as a predictor is notable.

**Table 2:** Utility of the predictive probability equation as a clinical diagnostic model in this study under 10-fold cross-validation compared with commonly used ILI criteria (for which no cross-validation is needed).

Variable	Sensitivity (%, and 95% CIs)	Specificity (%, and 95% CIs)	PPV (%, and 95% CIs)	NPV (%, and 95% CIs)	Overall accuracy (%, and 95% CIs)
Predictive probability equation, maximising total sensitivity and specificity	65(58, 72)	69(62, 75)	43(39, 47)	85(83, 87)	68(64, 71)
Predictive probability equation, maximising accuracy	18(8, 29)	96(93, 99)	67(57, 76)	77(75, 80)	76(74, 77)
Predictive probability equation, setting sensitivity to 90%	90(89, 90)	26(20, 23)	30(28, 33)	86(83, 89)	43(38, 48)
Fever ≥37.8°C, cough or sore throat	84(78, 83)	36(34, 38)	34(31, 35)	84(80, 85)	48(47, 51)
ILI (Fever ≥38.0°C, cough or sore throat)	69(64, 71)	55(53, 57)	37(35, 40)	81(79, 83)	58(57, 60)

## Discussion

Differentiating between influenza infections and other febrile respiratory illnesses is a challenge in clinical settings without laboratory assistance. In most situations, it is not feasible or cost-effective to perform PCR tests, while cheaper rapid tests have limited sensitivity [17], [18]. It is therefore important for clinicians to have clinical presentation-based guides to assist in diagnosing influenza cases for treatment and further management, especially during an epidemic or pandemic.

Influenza-positive and negative cases had several differing clinical parameters. We have found that influenza-positive cases were more likely to have running nose compared to influenza-negative cases, similar to the findings from another general population study in the tropics [7]. This is contrary to previous belief that running nose is less common in influenza compared to other viral respiratory illnesses [19]. Likewise, influenza cases also had similar prevalence of cough with sputum compared to non-influenza cases, also contrary to previous belief [19].

At the same time, influenza cases were more likely to have higher temperature and chills and rigors but less likely to present with sore throat, providing supporting evidence to a previous study by Monto and colleagues that one of the most predictive symptoms of influenza is fever [20]. However, unlike that study, we did not find that cough was a predictive symptom for influenza. Possible reasons for such a difference include the potentially different aetiologies for non-influenza cases in the tropics and other regions, and also possible differences in influenza presentation by region. It is therefore important to validate these predictive tools in the local setting where they are used.

In the absence of laboratory testing, using our clinical diagnostic model enabled accurate classification of up to 76% of all cases in our cohort (Table 2). Keeping sensitivity at 90%, we were able to achieve a high negative predictive value of 86%, which is useful for clinicians in excluding influenza cases. The positive predictive value, on the other hand, is low due to the substantial overlap in symptoms between influenza and non-influenza cases. The clinical diagnostic model performed significantly better than standard ILI criteria among our subjects with febrile respiratory infections. It can be easily adapted into various tabular or electronic formats for easy use by clinicians. This, if taken together with specific policy and cost evaluations in the local setting, may help guide initiation of anti-viral treatment or isolation measures during an epidemic or pandemic situation while reducing wrong treatment of non-influenza cases to minimize stockpile wastages.

The strengths of our study are its large sample size, high follow-up rate, and high diagnostic ascertainment, with etiological confirmation of all positive influenza cases. There are some limitations to this study, including the natural bias towards febrile symptomatic cases due to the case definition. Influenza cases do present with mild or asymptomatic infection, but these cases will be difficult to identify in a surveillance program and are less severe in clinical outcome. The results should therefore be interpreted in the context of febrile symptomatic infection requiring physician consultation, which capture the more severe and important cases that affect absenteeism.

In addition, this study predominantly considered young male adults. While we felt that there is no evidence that shows any differences in presentation by gender, further studies are required to determine if similarly high diagnostic ascertainment can be achieved in other age groups. Similarly, consultation biases may exist as the military population have medical consultation patterns that differ from the general population. We re-emphasize that diagnostic tools should be developed in the setting where they are used. Other potential biases include presentation biases from cases which rejected recruitment, presentations after recruitment hours which were not included, and losses to follow-up. Recall biases may exist as we obtained final clinical history two weeks after enrolment into the study, which we felt struck a balance between the risk of recall bias and the desire to capture comprehensively all symptoms during the illness period.

Different diagnostic scores may need to be developed to account for local FRI aetiologies and socio-cultural-demographic differences, but so doing will rely on well-designed local surveillance programs. The best clinical syndrome to be used for surveillance is a potentially interesting question that may be explored by further related studies.

Use of a predictive equation as a clinical diagnostic model can help better predict influenza than the conventional influenza-like illness definition among young adult military personnel with febrile respiratory illnesses. Until cheap, rapid and reliable point-of-care tests become widely available, clinical scores derived from large cohort studies may be of reasonable clinical utility.
